# Psychiatry

**Published:** 1905-12-02

**Authors:** 


### Progress in Medicine and Surgery.

### PSYCHIATRY.

(*Continued from page* 134.)

**Mental Unsoundness in Local (as distinct from Convict) Prisons.**—Dr. William Cotton, medical Officer of H.M. Prison, Bristol,[2] states that, excluding debtors, a local prison is one containing "persons charged with an offence awaiting trial, or convicted and serving a sentence of imprisonment of less than two years." All prisoners sentenced to penal servitude are—while awaiting their trial, or for a short time after conviction and sentence—liable to come to the local prison and so be within the cognisance of the medical officer there. All such prisoners are practically under continuous observation by day and night, and, if necessary, are placed in the hospital of the prison if ill. Under these conditions every opportunity is afforded to the prison physician to become acquainted with any symptoms of mental anomaly or aberration which may exist in any prisoner. As regards the mental condition of such criminals, with an alien or partially insane mind, says Dr. Cotton, four fairly natural groups may be distinguished according to symptoms.

(*a*) The first group consists of sane and (at least when on duty) temperate prisoners, whose mental condition and general physique is above the average condition of the outside world. Most of these are the real professional criminals who have calculated the chances and taken the risks, e.g. master burglars, master swindlers and organisers of swindles, forgers, the whole tribe of sharpers and confidence tricksters, fraudulent bankrupts, and the like, "acute, subtle, plausible, shameless, audacious, and often possessed of winning manners. All these gentry are pretty numerous in a local prison while waiting their trial at Quarter Sessions and Assizes. . . Some of the most admirable psychological studies of the great Victorian novelists are descriptive of individuals of this class, not to speak of later and more sensational writers."

(*b*) It is among the tribe of false-pretence men—sharpers, confidence tricksters, etc.—that the prison medical officer meets the most ingenious, and, it may be added, successful malingering. "Some of them simulate insanity itself most skilfully, and others more crudely. . . Then, again, it is here that the feigned attempts at suicide—the 'dinner-hour' suicide—are made. The *modus operandi* is generally the same. . . At the hour of collecting the tins (from which the prisoners dine), when the discipline staff are sure to be more numerously about, or after ringing his bell, a prisoner is presently found blue in the face, suspended by his handkerchief to a hook in the wall, or else lying on the floor with a strand of oakum twisted tightly round his neck. . . A successful suicide in a locked cell would be terrible to think of, and implies . . . a case of insanity overlooked. Yet from what I have seen of seven or eight feigned attempts it seems to me that most of them were very narrow escapes from death by misadventure." Subsequent confession in these cases left no doubt of their feigned nature. Discipline, however, in local prisons now is said to be more severe than in a convict prison, so that these and other "malingerers," who have reason to know, prefer three years' penal servitude to two years in a local prison.

(*c*) The group of prisoners with symptoms and signs of inebriety forms the largest in the local prison, "constituting probably 90 per cent. of the males, and even a larger proportion of the females, received into Bristol prison. It consists of men and women . . . who, through drunkenness and its associated follies, have become the subjects of a criminal charge, or in whose offence drink has been (in the wider sense) a contributing cause . . . and who, when the drink is in them, are for a time practically more or less insane." In the surroundings of a prison they are, or soon become, sober, and probably would continue so in a nonalcoholic place. Very many of them are alcoholic recidivists, and in time, no doubt, become progressively deteriorated, and finally may develop the symptoms of chronic alcoholic dementia. There is no crime, however serious, that members of this group may not commit, and it is notorious that to their account belong most cases of insane violence; in this group of cases typical delirium tremens of great severity may occur.

(*d*) At the lower end of the scale are a group of (generally habitual) petty offenders, who are just as obviously under the general average of physique and mental ability as the others—great professional criminals are constitutionally above it. These petty criminals, approximate to the type of the "neurasthenic congenital criminal," described by the eminent alienist physician of the State Prison of Vienna—Professor Moritz Benedikt.[3] They are rather asocial than actively or dangerously anti-social, and find their degenerate counterparts (at large) in tramps and vagrants (male branch) and prostitutes (female branch). "From bad heredity, fostered by a bad environment, they have little or no chance," says Dr. Cotton, "in the struggle for social survival . . . being poorly nourished and clothed, mere social flotsam and jetsam, and hardly likely to do much in life except propagate their kind."

(*e*) Finally, the last group consists of a motley collection of congenitally weak-minded "moral imbeciles," a class dangerous to themselves and others when out of control, and some youthful cases of epileptic insanity and mild dementia præcox, whose proper home should be the asylum, or (in the milder cases of youthful epilepsy with a moderate degree of sanity) the life and environment of a "farm colony," such as Chalfont St. Peter. The problem of the practical care and treatment of this type of criminal seems to be realised at the present day. In the discussion which followed the reading of the above contribution at the meeting of the Medico-Psychological Association (S.W. Division) in 1904, Dr. Weatherby said that Dr. Cotton's paper pointed out clearly what he (Dr. Weatherby) had been maintaining for many years—that many who went to prison ought never to go there, but rather to go straight from the Court to an asylum, to remain there for practically the rest of their lives under care and treatment. Dr. Cotton had shown that he got a large number of prisoners who came up over and over again for crimes they ought never to have been allowed to commit. Dr. Stewart (Bridgend) stated that his own experience as an asylum physician agreed with Dr. Weatherby that many of such prisoners were irresponsible. When present at Assizes it seemed to him deplorable that judges, barristers, and jurists should be engaging in dealing with half-imbecile prisoners who came up time after time. Dr. J. Stewart (Clifton) said that they, as alienists, who went deeper into the symptoms of morbid psychology than the general public, could see "what the general public could not see, and what they would not believe if they were told, that there were a number of people poorly born, poorly bred, poorly fed, and in every way unfavourably circumstanced (as regards mental health) among the habitual offenders." Dr. Cotton concluded by showing how, while some cases of the five or six types he dealt with were fairly suitable for local prison treatment, especially where the resident physician in charge had experience of the insane, that in a considerable proportion of cases the asylum was the right home for these anti-social failures.

[2] Jour. of Ment. Sci., January, 1905. [3] *Vide* a reference to and description of this in the Jour. of Ment. Sci. in April, 1899, in the article on the Psychology and Classification of Mental Diseases.

